# Low vertebral CT Hounsfield units: a risk factor for new osteoporotic vertebral fractures after the treatment of percutaneous kyphoplasty

**DOI:** 10.1007/s11657-022-01177-8

**Published:** 2022-10-29

**Authors:** Kaifeng Ye, Da Zou, Fang Zhou, Weishi Li, Yun Tian

**Affiliations:** 1 Department of Orthopaedics, Peking University Third Hospital, Haidian District, No. 49 North Garden Road, Beijing, 100191 China; 2 Engineering Research Center of Bone and Joint Precision Medicine, Ministry of Education, Beijing, China; 3 Beijing Key Laboratory of Spinal Disease Research, Beijing, China

**Keywords:** Osteoporotic vertebral compression fracture, HU value, Re-fracture

## Abstract

**Purposes:**

To identify the characteristics of the vertebral HU in the elderly patient with new osteoporosis vertebral compression fractures (OVCF) after treatment of percutaneous kyphoplasty (PKP), which may help us to preliminarily evaluate the risk of a new OVCF after the treatment of PKP.

**Methods:**

We retrospectively analyzed the patients who received PKP treatments in our hospital to find out the patients suffered new OVCFs after the treatment of PKP and set an age-, sex-, first fracture vertebrae-, surgical segment-, and comorbidity-matched control group without new fractures. We measured the axial and sagittal L1-HU values to compare their differences.

**Results:**

There were 32 patients who suffered new OVCFs and received another PKP surgery in our department. In the study group, the average L1 sagittal and axial HU values were 46.17 ± 21.31 HU and 47.77 ± 22.38 HU, and they had no statistical difference (*P* > 0.05). For the control group, the average L1 sagittal and axial HU values were 75.69 ± 29.72 HU and 80.23 ± 30.26 HU, and their difference was not significant (*P* > 0.05). No matter from the axial or sagittal evaluation, the L1 HU value in the study group was significantly lower than that in the control group (*P* < 0.001). The AUC of using the L1 axial HU value to differentiate patients with new fractures from controls was 0.85 while the sagittal one was 0.82. In axial (and sagittal) evaluation, the cutoff value (adjusted to the multiple of five) had high specificity of 90% or high sensitivity of 90% to identify patients with new fractures of 45 HU and 75 HU (50 HU and 75 HU), respectively.

**Conclusions:**

The lower the vertebral HU value is, the more likely the patients suffer new OVCFs after PKP treatment.

## Introduction

Osteoporosis is a worldwide problem annoying the elder people and is recognized as a progressive reduction in bone mineral density (BMD). According to the epidemiological investigation, it affects almost 30% of the elderly, especially for postmenopausal women [1, 2]. Fractures due to osteoporosis have become a global public issue, it is estimated that about 40% of women over the age of 50 years will suffer from osteoporotic fractures during their lives [3]. The most common type of osteoporosis fracture is osteoporosis vertebral compression fractures (OVCFs), the estimated annual cases of OVCFs in China is around 1,110,000 [4]. Patients with OVCFs will suffer from pain, damaged physical function, lower quality of life, and even death, which leads to high health and social burden [5, 6]. Percutaneous kyphoplasty (PKP) is a common treatment for OVCFs, which could quickly relieve the pain and reinforce the fractured vertebrae to strengthen the spinal stability [7]. However, several studies have reported the new-onset OVCFs in patient treated by PKP. The incidence of new-onset thoracolumbar vertebral fracture after PKP ranged widely from 2 to 23% [8–10]. New fractures could also bring severe pain, spinal deformity, and deprivation of daily activities; the treatments were an extra burden to the affected patient and society as well [11, 12].

Previous studies have reported several possible influencing factors of re-fractures after PKP, such as BMD, previous fracture history, back muscle strength, and biomechanical change, and low BMD of the vertebral body was the most recognized risk factor [8–11, 13, 14]. For the assessment of BMD, the recommended gold standard by WHO is the dual-energy X-ray absorptiometry (DXA) scanning. However, many studies had stated the low popularity of the DXA test and its disadvantage of overestimation [15–20]. Moreover, due to the severe pain effect of OVCFs and the limitation of emergency department condition, OVCF patients were unable to perform a DXA test. The above problems limit our further evaluation of the risk of new OVCFs after PKP treatment in clinical practice.

Pickhardt [21] et al. first figured that the CT attenuation values have high correlation with the result of DXA. Thereafter, many researchers also proved that the use of CT value could be an alternative method for opportunistic screening of osteoporosis [19, 20, 22, 23]. Our previous studies are consistent with them and figure out that the lower the vertebral HU value is, the more likely the patients have more than one vertebral fracture [24, 25].

Taking the serious complications of new fractures and the deficiency of the DXA test together into consideration, our study aims to figure out the characteristics of the vertebral HU in the elderly patient who come up with a new OVCF after the treatment of PKP, which may help us surgeon to preliminarily evaluate the risk of a new OVCF of osteoporotic patients after the treatment of PKP.

## Patients/methods

### Patient cohort

This study was a retrospective analysis, and approval was obtained from the Institutional Ethics Committee of the Peking University Third Hospital. All the patients that were selected from our database of the OVCFs patient accepted PKP surgery in our hospital from January 2012 to December 2020. The inclusion and exclusion criteria were as follows.

- Inclusion criteria:
  1. patients aged 55 or older,
  2. underwent PKP surgery for OVCF caused by low-energy trauma,
  3. performed spine CT examination in our hospital within 1 month before the first PKP surgery, and
  4. the new fractures were caused by low-energy trauma.

- Exclusion criteria:
  1. OVCFs caused by high-energy trauma;
  2. pathological fracture
  3. patient with history of spine surgery;
  4. patient treat by percutaneous vertebroplasty
  5. patient had fractures at each vertebra of T12-L2, which were unable to determine the L1-HU value.

To identify the difference of the L1-HU value between the groups with or without new OVCFs after the treatment of PKP, an age-, sex-, first fracture vertebrae-, surgical segment-, and comorbidity-matched control group without new fractures was chosen from the same database. The age tolerance limit we set was 2 years while the surgical segment tolerance limit was 1 level vertebrae, and we successfully matched 32 patients without new OVCFs.

### Treatment protocol

In the emergency department or outpatient department, for patients with suspicion of OVCFs, our doctors arranged X-ray, CT, and MRI examinations to identify the fractures. The confirmed fractured patients were admitted to the hospital. Each of the cases was included in pre-operative discussions by more than four experienced orthopedic trauma surgeons for surgical procedures. The operations were conducted by skilled surgeons under the condition of general or local anesthesia and fluoroscopy control on a radiolucent fracture table. After satisfy anesthesia, two small incisions are made bilateral to the pedicles, and the two cannulas were docked onto the pedicles. Under strict guidance by fluoroscopy, the cannulas were advanced into the vertebral body via pedicles. The balloon was inserted and inflated to restore the vertebral height. The ropy cement was slowly injected into the body under strict guidance until the cement approached the posterior wall and finally sutured the incisions. All the patient after PKP treatment would start their anti-osteoporotic drug treatment procedures in a routine scheme, and the routine medicines were calcium tablet (600 mg, oral administration, once a day), calcitriol (0.25 ug, oral administration, thrice a day) and salmon calcitonin (200 IU, nasal spray, once a day). During the treatment period, we need to monitor the serum calcium level once a month to adjust the medicine dose.

### Vertebral HU

**Fig. 1:**
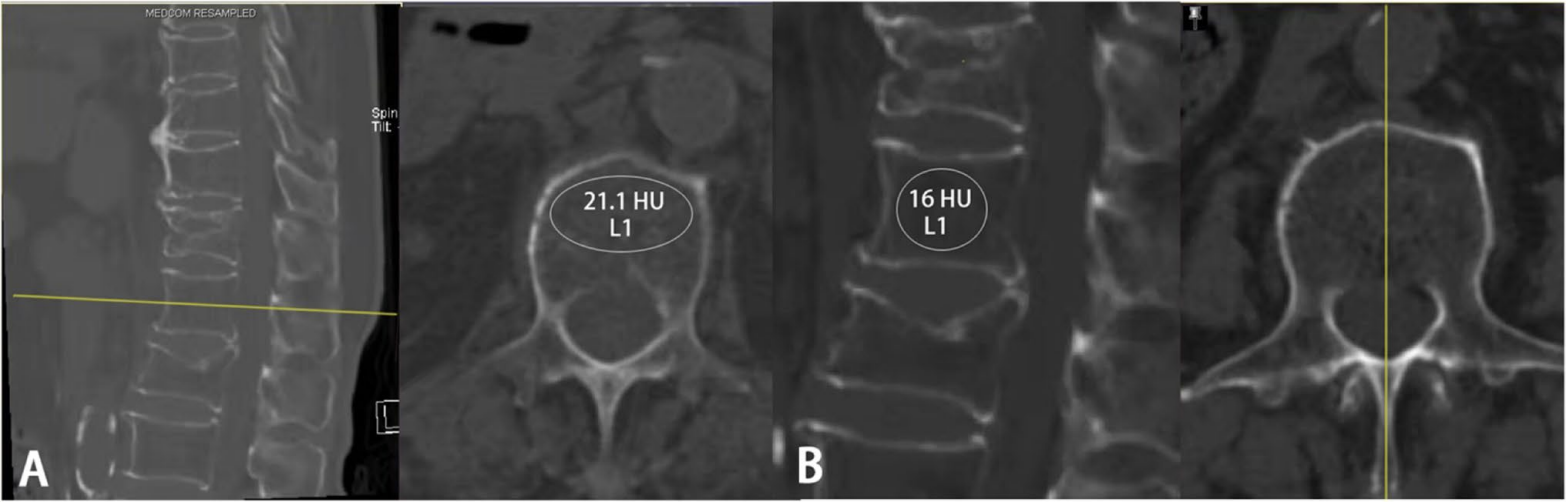
Example of CT HU measurement: when drew an oval region of interest (ROI) over the axial (**A**) and sagittal (**B**) of L1 mid-body, PACS software automatically calculates the average CT HU for the region of interest. These were the CT images of a 77 years old female patient who suffered a T12 OVCF; the axial and sagittal L1-HU values were only 21.1 HU and 16 HU, respectively. She suffered a second fracture of L1 after only 2 months

In this study, the research index is the vertebral HU. As the studies mentioned in the “Introduction,” the L1 vertebral body was chosen for HU measurements with preoperative three-dimensional reconstructive spine CT (Siemens, DEFINITION, tube voltage 120 kV). The CT scanners were daily calibrated during the period of time. CT HU value is the corresponding value of each tissue equivalent to the X-ray attenuation coefficient in CT image, it ranged from − 1000 to 1000 HU (air: − 1000 HU; water: 0 HU; cortical bone: 100 HU). Like the previous studies [19–25], we used the picture archiving and communication system (PACS) to measure HU value. Putting the oval region of interest (ROI) on the middle-axial and middle-sagittal image within the trabecular bone of the L1 vertebral body to calculate the average CT HU value (Fig. 1). If the L1 vertebrae were fractured, the average HU value of T12 and L2 was used as the alternative for L1-HU. If there were fractures both at T12 and L1, the L2-HU would replace the L1-HU. If there were fractures both at L1 and L2, the L1-HU was replaced with the T12-HU. The cases came up with fractures at each vertebra of L1, L2, and T12 were excluded.

### Statistical analysis

All the statistics in this study were analyzed using SPSS (version 24). We used the case–control matching function of SPSS to find the age-, sex-, first fracture vertebrae-, and surgical segment-matching control group. The continuous data was tested for the normality by the Shapiro–Wilk test. Student’s *t*-test was used to analyze the data obey to normal distribution; the Mann–Whitney *U* test was performed to test the non-normal distribution data. Receiver operating characteristic curve analysis (ROC) and the area under the curve (AUC) were used to evaluate the performance of using HU value to distinguish patients with new fracture from control patients.

## Results

In total, there were 987 patients who received PKP treatment in our hospital, but only 561 of them had CT examination. There were 36 patients who suffered new OVCFs and received another PKP surgery, but 3 were pathological fracture, and 1 had vertebral fractures in T12, L1, and L2, and they were excluded. For the included 32 cases, the average age was 74.25 ± 8.08 years (range from 56 to 88 years), and the average visual analogue score (VAS) was 7.19, and there include 3 men and 29 women (Table 1). Eleven of them had the comorbidity of hypertension, 6 had diabetes, and 6 had coronary heart disease. The first fractured segments of these 32 cases involved 33 vertebral fractures, including 2 cases of T7, 3 cases of T8, 2 cases of T11, 8 cases of T12, 5 cases of L1, 8 cases of L2, 1 case of L3, 1 case of L4, 1 case of L5, and 1 case of L4 and L5. The average interval time between the first fractures and the new OVCFs was 9.25 months (1 to 36 months). Half of the new fractures involved the thoracic vertebrae and half involved the lumbar vertebrae. Meanwhile, 15 new fractures occurred in the vertebrae adjacent to the cemented segments, and the rest was not. Moreover, 8 patients had suffered more than one new fracture. There were 5 patients who had L1 fracture; their L1 HU were replaced with the mean HU value of L2 and T12.

**Table 1:** The descriptive analysis of two groups

	Gender (man/woman)	Age	VAS
Study group	3/29	74.25 ± 8.08	7.19
Control group	3/29	74.47 ± 8.24	7.09
*P*	1.000	0.915	0.622

For the 1:1 age-, sex-, first fracture vertebrae-, surgical segment-, and comorbidity-matched control group, we successfully matched 32 patients without new OVCFs, including 3 men and 29 women, which was consistent with the study group. The average VAS was 7.09, which had no statistical difference with the study group. The average age was 74.47 ± 8.23 years (range from 57 to 88 years), which was close to the study group and without a statistical difference (*P* = 0.915) (Table 1). The situations of first fracture vertebrae, surgical segment, and comorbidity were all consistent with the study group. The normality of the L1 sagittal and axial HU values of two groups were tested by the Shapiro–Wilk test, and the data of sagittal HU value in the control group (*P* < 0.05) was abnormally distributed while the rest was normal (*P* > 0.05) (Table 2). In the study group, the average L1 sagittal HU value was 46.17 ± 21.31 HU (− 21.2 to 77.4 HU) while the average axial one was 47.77 ± 22.38HU (− 9.8 to 96.5 HU), and there was no significant difference between them (*P* > 0.05). In the control group, the average L1 sagittal HU value was 75.69 ± 29.72 HU (15.5 to 184.6 HU) while the average axial one was 80.23 ± 30.26 HU (14.1 to 171.2 HU), and their difference was no significant (*P* > 0.05) (Table 3).

**Table 2:** The Shapiro–Wilk test for normality of two groups

Groups		Statistic	*df*	*P*
Axial	Study group	0.978	32.000	0.752
	Control group	0.949	32.000	0.139
Sagittal	Study group	0.939	32.000	0.071
	Control group	0.887	32.000	0.003

**Table 3:** The comparison of L1 vertebral HU value of two groups

	Study group	Control group	*t*/*Z*	*P*
Axial-HU	47.77 ± 22.38 HU	80.23 ± 30.26	4.880	< 0.001^*^
Sagittal-HU	46.17 ± 21.31 HU	75.69 ± 29.72	− 4.196	< 0.001^#^
*t*/*Z*	0.650	− 1.580		
*P*	0.520^*^	0.114^#^		

^*^Student’s *t*-test.

^#^Mann–Whitney’s *U* test.

**Fig. 2:**
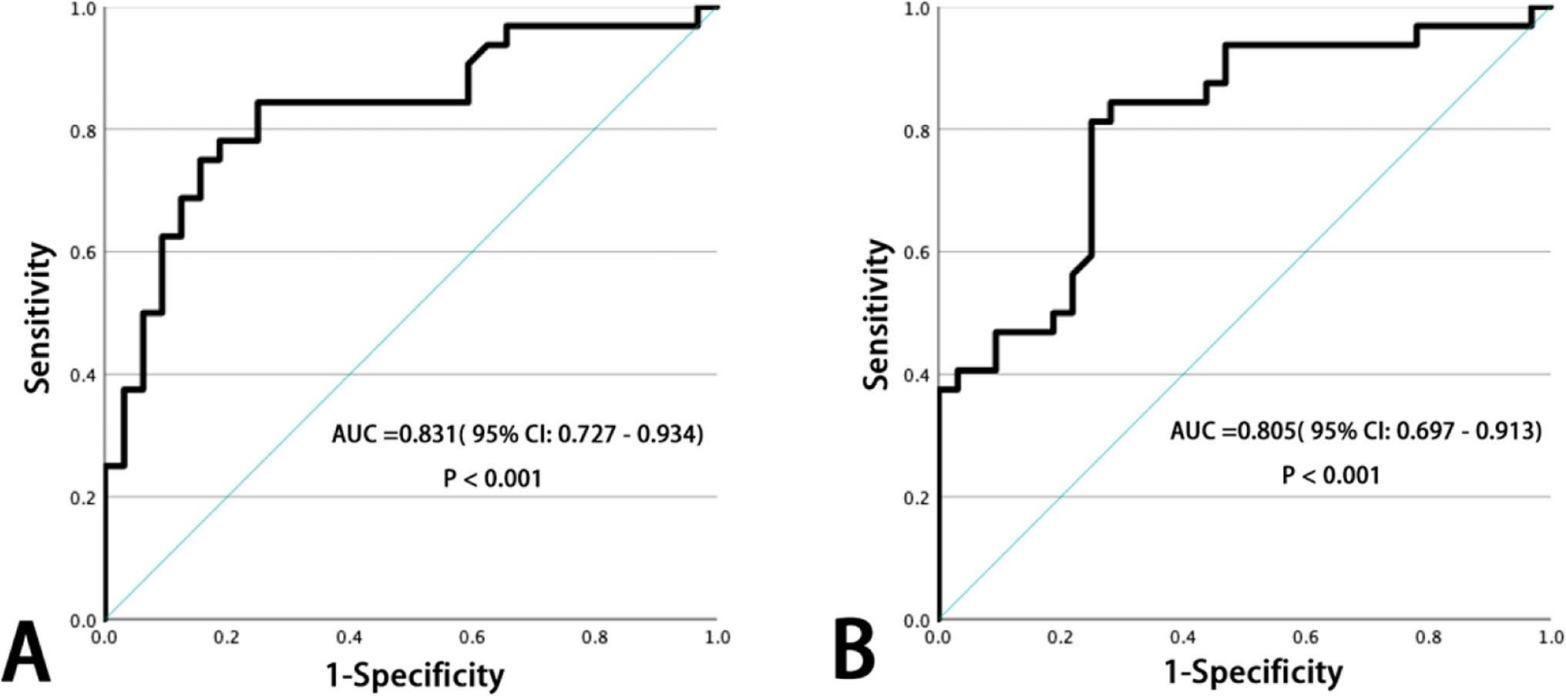
The AUC of using L1 axial (**A**) and sagittal (**B**) HU values to differentiate patients with new fractures from the control group

For the comparison between the study and control groups, no matter from the axial or sagittal evaluation, the L1 HU value (mentioned above) in the study group was significantly lower than that in the control group, the student *t*-test for axial comparison and the Mann–Whitney *U* test for sagittal comparison turned out that the *P* value were both lower than 0.001 (Table 3). The AUC of using L1 axial HU value to differentiate patients with new fractures from controls was 0.831 (95% CI 0.727–0.934, *P* < 0.001) while the sagittal one was 0.805 (95% CI 0.697–0.913, *P* < 0.001) (Fig. 2). In axial evaluation, the cutoff value (adjusted to the multiple of five) had high specificity of 90% or high sensitivity of 90% to identify patients with new fractures of 45 HU and 75 HU, respectively. In sagittal evaluation, the cutoff value (adjusted to the multiple of five) had high specificity of 90% or high sensitivity of 90% to identify patients with new fractures of 50 HU and 70 HU, respectively.

## Discussion

Our study expressed that the patients who come up with new OVCFs after the treatment of PKP are characterized by much lower vertebral HU values than those without, and both the sagittal and axial L1-HU values can be the point to identify these high-risk population. Therefore, in our clinical practice, we should pay more attention to the patients with low L1-HU value, we need to strongly emphasize the importance of the subsequent anti-osteoporotic drugs treatment, muscle exercise, and personal protection after the PKP treatment.

As early as in 2011, Pickhardt [21] had raised the idea that vertebral HU was an alternative method to evaluate the bone quality without additional cost and radiation. After that, many researchers carried out their studies, and all proved that the measurement of the vertebral HU value was a good approach to detecting osteoporosis, and the measurement of L1 vertebrae was the optimal choice. Our previous study [25] demonstrated that the elderly patients with acute OVCFs were characterized by much lower vertebral HU values than those without. Furthermore, we divided osteoporotic patients into three categories based on L1-HU values: HU value of 80–110 for mild osteoporosis, HU value of 50–80 for severe osteoporosis, and HU value of ≤ 50 for extremely severe osteoporosis meaning high risk of ≥ three levels of vertebral fractures. These are the preliminary work for this study.

Many studies [9, 11] had focused on the risk factor of the new fractures following the primary OVCFs, but none of them put their attention to the relationship between new fractures and L1-HU values after PKP treatment. The most acceptable risk factor was low BMD [9, 11, 12, 26]. However, as we mentioned above, the accuracy of the DXA test was insufficient, and many patients did not perform a DXA test before the fractures occurred; furthermore, the further DXA test was limited under the condition of emergency department, which limited our further evaluation in clinical practice. From literature studies [22–25], we realized that the vertebral HU value was an alternative method to evaluate bone quality. Therefore, our study was performed, and trying to clarify the relationship between new fractures after PKP treatment and L1-HU value, we established the age-, sex-, first fracture vertebrae-, surgical segment-, and comorbidity-matched control group and found out that the patients in the study group had much lower L1-HU value than the control group. And the AUC of ROC analysis displayed that the L1-HU value had excellent performance in differentiating new fractures, which suggested that the lower the L1-HU value was, the more possibility of coming up with a new fracture. The low vertebral HU value suggests the low density of vertebral trabecular distribution, which was consistent with that the low BMD was a risk factor of new fractures. Our outcomes also demonstrated that there was no significant difference between the evaluation between sagittal HU value and axial one, which was consistent with the opinion with Pickhard; he suggested that both sagittal and axial trabecular attenuation showed good correlation with DXA results, and the two measurement methods had no systematic bias [27].

In our study, the AUC of using L1-HU values to identify patients came up with new OVCFs after PKP treatment was over 0.80 (sagittal: 0.805; axial: 0.831), stating that the L1-HU values were an excellent marker to identify the high-risk population during our first surgical treatments. For instance, the cutoff sagittal and axial HU values which had high specificity of 90% to identify patients with new fractures were 50 HU and 45 HU respectively. Therefore, in our clinical practice, we should keep an eye on the patients with sagittal L1-HU value ≤ 50HU (or axial HU value ≤ 45HU); we need to inform the patients of the high risk of suffering with new OVCFs and strongly emphasize the importance to prevent the subsequent fractures. For these patients, we should start their anti-osteoporotic drug treatment and keep follow to ensure their medicine compliance; they should receive standard anti-osteoporotic treatment. Furthermore, patients themselves should keep their mind to be more careful in daily life; they should protect themselves from unnecessary trauma, which is the direct cause for subsequent OVCFs. Recently, Deng [13] et al. found that postoperative back muscle exercise could effectively maintain the bone density and further reduce the risk of refracture.

Our study is the first one to explore the relationship between the new fracture after PKP treatment and CT value. However, there were some limitations. First, the database we possessed was the patients who received surgery in our hospital, there existed some patients who suffered new OVCFs, but they did not come back for treatment or looked for surgery in other hospitals. As a result, the total number of patients who suffered new OVCFs we studied was less. Second, the diversity of clinical CT scans may have an impact on the vertebral HU due to the influence of scanning parameters and software; therefore, the CT machine must be calibrated before the scanning. Thirdly, nearly half of the patients in our database had no CT examination and were excluded for this study, which may have selective bias; therefore, a prospective study controlling selection bias is needed in the future.

## Conclusions

The vertebral CT HU value is an excellent predictor for new fractures of patients who received PKP surgery. No matter we evaluate the sagittal or axial HU value, the lower the vertebral HU value is, the more likely the patients suffer new OVCFs after PKP treatment. In our clinical practice, if the L1 vertebral HU value is lower than 45 HU in axial or 50 HU in sagittal evaluation, we need to pay more attention to these patients and emphasize the importance of preventing new fractures.
